# Analysis of Pulsatile Vessel Expansion in Healthy, COPD‐ and PH‐Patients Using Dynamic Vessel Segmentation in Free‐Breathing Lung MRI

**DOI:** 10.1002/jmri.70249

**Published:** 2026-01-31

**Authors:** Julian Glandorf, Marius M. Klein, Filip Klimeš, Agilo Luitger Kern, Marcel Gutberlet, Jens M. Hohlfeld, Marius M. Hoeper, Karen M. Olsson, Frank Wacker, Jens Vogel‐Claussen, Andreas Voskrebenzev

**Affiliations:** ^1^ Institute for Diagnostic and Interventional Radiology Hannover Medical School Hannover Germany; ^2^ Biomedical Research in End‐Stage and Obstructive Lung Disease Hannover (BREATH), German Centre for Lung Research (DZL) Hannover Germany; ^3^ Department of Diagnostic and Interventional Radiology Charité Universitätsmedizin Berlin, Corporate Member of Freie Universität Berlin and Humboldt‐Universität Zu Berlin Berlin Germany; ^4^ Clinical Airway Research, Fraunhofer Institute for Toxicology and Experimental Medicine (ITEM) Hannover Germany; ^5^ Department of Respiratory Medicine and Infectious Diseases Hannover Medical School Hannover Germany

**Keywords:** COPD, delay, lung MRI, pulmonary hypertension, pulmonary vasculature, vessel expansion

## Abstract

**Background:**

The pulsatile expansion of pulmonary vessels carries dynamic cardiopulmonary information that may reveal disease earlier than structural changes alone.

**Purpose:**

To test (i) intra‐ and inter‐scan repeatability of dynamic vessel metrics in healthy subjects, and (ii) whether chronic obstructive pulmonary disease (COPD) and postcapillary pulmonary hypertension (PH) exhibit disease‐specific differences.

**Study Type:**

Retrospective, multi‐cohort feasibility study with repeatability sub‐study.

**Subjects:**

Healthy: 29 (11 female), COPD: 52 (15 female), PH: 25 (15 female) with isolated postcapillary PH.

**Fieldstrength/Sequence:**

1.5T, free‐breathing spoiled gradient‐echo sequence, TE = 0.82 ms, TR = 3 ms, flip angle = 5°.

**Assessment:**

2D U‐Nets segmented pulmonary vessels throughout each series of 250 images. Dynamic metrics included the coefficient of variation (CV) of vessel area and CV of vessel signal. Delay between vessel signal and vessel expansion as %‐of‐RR‐interval (SE‐delay) together with the heart rate and lung area were calculated.

**Statistical Tests:**

Repeatability: Friedman; intraclass correlation coefficient (ICC); standard error of measurement (SEM) and the minimal detectable change (MDC). Group comparisons: Kruskal–Wallis and multiple linear regression. *p* < 0.05 was considered significant.

**Results:**

Only the heart rate and the SE‐delay presented significant changes across repetitive scans. Differences in Bland–Altman plots were evenly distributed across the range of measurements and symmetrically scattered around zero within 95% confidence intervals (mean bias 0.08%–5.12%). Across cohorts, CV vessel area and CV lung area differed significantly, showing lowest variability in COPD (2.67%; 7.03%) and highest in PH (4.58%; 16.74%). SE‐delay was significantly prolonged in COPD (81.98%) compared to PH (67.20%) and healthy participants (62.97%). Cohort status (Healthy/COPD/PH) remained the strongest significant predictor for all parameters after adjustment (*F*‐values 4.62–34.04), while age and gender had no significant influence (*p* > 0.391; *p* > 0.069).

**Data Conclusion:**

Free‐breathing lung MRI with automated vessel segmentation reveals distinct hemodynamic characteristics in COPD and PH. This method shows potential as a sensitive non‐invasive tool for detection, phenotyping, and treatment monitoring in pulmonary pathology.

**Evidence Level:**

3.

**Technical Efficacy:**

2.

## Plain Language Summary

Diseases of the lungs and heart can change how blood vessels expand with each heartbeat, often before structural damage is visible. We conducted this study to investigate whether these subtle vessel movements can be measured reliably using lung MRI and whether they differ between healthy individuals and patients with chronic obstructive pulmonary disease (COPD) or pulmonary hypertension (PH). Using automated image analysis, we tracked changes in lung vessels over time. We found that vessel behavior differed clearly between disease groups. These findings suggest that this MRI‐based approach could become a useful tool for detection, classification, and monitoring of lung diseases.

## 1 Introduction

In contrast to alveoli, the extensive vascular network within the lungs offers excellent potential for proton MRI. Blood vessels provide sufficient signal due to the high spin density of blood and enable the exploitation of flow effects for functional analyses [1, 2, 3]. Moreover, their structure is closely linked to the functional capabilities of the lungs themselves [4]. Importantly, dynamic information from the cardiovascular system has proven to be highly valuable, as it can reflect functional changes within this complex cardiorespiratory interplay early or even before structural defects become apparent [5].

Chronic lung diseases in particular lead to remodeling and destruction of the pulmonary vasculature. As the disease progresses, pulmonary vascular resistance increases to the extent that mean pulmonary arterial pressure at rest exceeds 20 mmHg, which defines pulmonary hypertension (PH) [6]. Over time, this strain may overwhelm the adaptive capacity of the right heart, resulting in dilation and/or hypertrophy and eventual development of cor pulmonale, accompanied by functional decline [7].

For instance, the combined effects of inflammation, hypoxia, and capillary loss due to severe emphysema increase pulmonary vascular resistance and pressure in chronic obstructive pulmonary disease (COPD) [8]. Additionally, cardiac output is reduced in COPD, presumably due to increased intrathoracic pressure caused by hyperinflation and hypoxic vasoconstriction, which diminish cardiac preload [9].

Beyond COPD, many other diseases affect the pulmonary vasculature and may cause PH [7, 10]. PH can be categorized based on the hemodynamic characteristics, depending on the location of the causative mechanism relative to the capillary bed [6]. While COPD predominantly leads to precapillary hypertension, left heart disease causes postcapillary hypertension, and other conditions may result in combined pre‐ and postcapillary hypertension. These forms are typically differentiated invasively by right‐heart catheterization and pressure measurements [6].

Numerous studies have investigated noninvasive methods to assess the hemodynamic properties of various lung diseases. In the proximal vasculature, phase‐contrast MRI and echocardiography can detect pulmonary arterial stiffness through reduced distensibility of the central pulmonary arteries in COPD [11, 12, 13]. At the capillary level, red blood cell (RBC) oscillations measured by xenon lung MRI have gained increasing attention [14, 15, 16]. Wang et al. demonstrated distinct xenon MRI signatures with varying RBC oscillation amplitudes among healthy individuals, COPD patients, and PH patients [17].

The aim of this study was to explore the quantitative characterization of the pulmonary vasculature using dynamic vessel parameters derived from non‐contrast MRI. Specifically, the study evaluated intra‐ and inter‐scan repeatability in healthy participants and examined parameter differences among healthy individuals, patients with COPD, and patients with isolated postcapillary PH.

## 2 Materials and Methods

### 2.1 Study Participants

**FIGURE 1:**
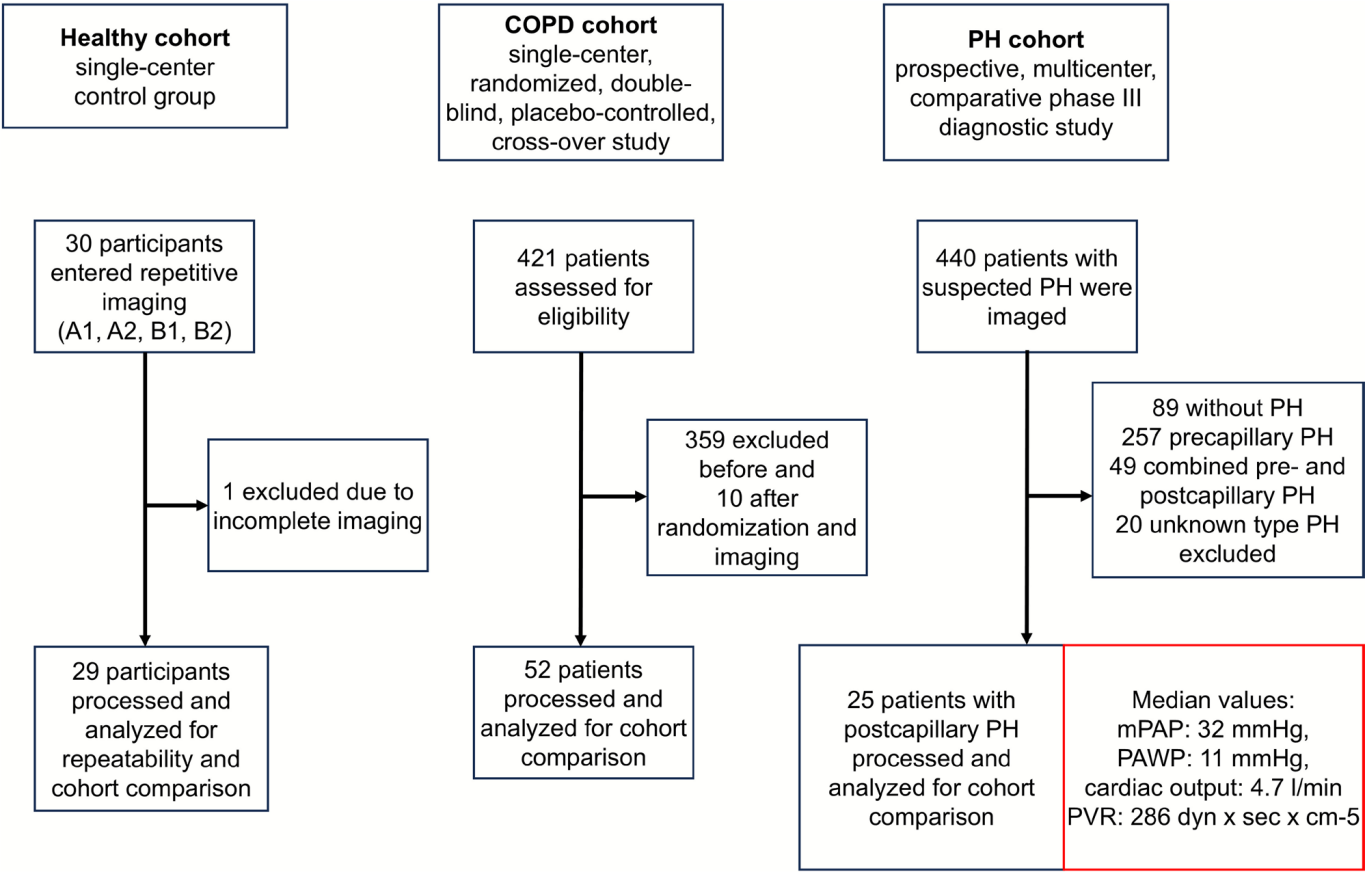
Data from three different studies were evaluated retrospectively for this study. For more detailed information see ClinicalTrials.gov identifiers NCT02442206 and NCT02791282. COPD, chronic obstructive pulmonary disease; mPAP, mean pulmonary artery pressure; PAWP, pulmonary artery wedge pressure; PH, pulmonary hypertension; PVR, pulmonary vascular resistance.

Data from three different studies were evaluated retrospectively for this exploratory study (Figure 1). All studies were approved by the institutional ethics committee. Two of the trials are registered at ClinicalTrials.gov under the identifiers NCT02442206 and NCT02791282. Written informed consent was obtained from all participants.

The healthy cohort consisted of 29 participants (Table 1). Each participant underwent two consecutive MRI sessions (A and B) with intra‐scan repetitions (A1 and A2; B1 and B2), with a break of approximately 45 min between A2 and B1, during which the participants left the scanner room. Identical imaging parameters were used across all scans [18].

**TABLE 1:** Demographical data of the healthy‐, COPD‐, and PH‐cohorts.

	Healthy cohort	COPD cohort	PH cohort	*p*
Male/Female, *n*	18/11	37/15	10/15	**= 0.032** ^a^
Age (years)	26 [25–29]	65 [63–68]	73 [55–78]	**< 0.001** ^b^
Height (cm)	181 [174–185]	170 [169–175]	165 [160–180]	**< 0.001** ^b^
Weight (kg)	80 [67–89]	85 [77–87]	80 [73–102]	< 0.261^b^
BSA (m^2^)	1.99 [1.83–2.17]	1.97 [1.87–1.99]	2.01 [1.74–2.24]	< 0.896^b^
Key inclusion criteria	No cardiovascular or pulmonary disease	Confirmed diagnosis COPD with 10 pack years, obstruction and hyperinflation in spirometry	Isolated postcapillary pulmonary hypertension	

*Note*: Data are presented as median [IQR]. Body surface area formula of Dubois was applied. Significant *p*‐values in bold.

Abbreviations: BSA, body surface area; COPD, chronic obstructive pulmonary disease; PH, pulmonary hypertension.

^a^ *χ* ^2^ test.

^b^ Kruskal–Wallis test.

The COPD cohort included 52 participants (Table 1). All were at least 40 years old and had a confirmed clinical diagnosis of COPD. Inclusion criteria required a smoking history of at least 10 pack‐years and baseline hyperinflation, defined as a residual volume exceeding 135% of the predicted value. Airflow limitation was confirmed by a post‐bronchodilator forced expiratory volume in 1 s (FEV_1_) of less than 80% of the predicted value and a post‐bronchodilator FEV_1_‐to‐ forced vital capacity (FVC) ratio of less than 0.7. Participants with stable cardiovascular disease were eligible, whereas those with arrhythmias, heart failure (left ventricular ejection fraction < 40%), unstable ischemic heart disease, or uncontrolled hypertension were excluded [9].

The PH cohort consisted of 74 patients (Table 1). Inclusion criteria were transthoracic echocardiographic evidence of pulmonary hypertension, clinical suspicion of chronic thromboembolic pulmonary hypertension, and scheduling for SPECT imaging. For this analysis, only patients with confirmed postcapillary pulmonary hypertension based on right‐heart catheterization were included.

Exclusion criteria for all participants included: age below 18 years, any contraindications for MRI (e.g., cardiac pacemakers, claustrophobia, hypersensitivity to intravenous contrast agents), and pregnancy or breastfeeding.

These patient cohorts have previously been reported [9, 18, 19, 20, 21]. The prior evaluations included cardiac parameters, ventilation‐/perfusion‐metrics, and pulse wave velocity measurements.

### 2.2 Imaging

Imaging was performed on 1.5T MRI systems (Magnetom Avanto or Magnetom Aera, Siemens Healthineers, Erlangen, Germany) using surface coils and a spoiled gradient echo sequence with the following parameters: field of view = 50 × 50 cm^2^, matrix size = 128 × 128 (128 × 96 in COPD cohort), slice thickness = 15 mm, TE = 0.82 ms, TR = 3 ms, flip angle = 5°, pixel bandwidth = 1500 Hz/pixel, and for the healthy‐ and PH‐cohorts parallel imaging with generalized autocalibrating partially parallel acquisitions (GRAPPA) and an acceleration factor of 2. The parameters led to a temporal resolution of 384 ms for the healthy‐ and PH‐cohort and 288 ms for the COPD cohort. All images were interpolated to a final in‐plane resolution of 1.95 × 1.95 mm^2^. All participants were scanned in head‐first supine positions. In total, 200 (COPD‐cohort) and 250 (Healthy‐ and PH‐cohort) images of a single coronal slice at the level of the carina were acquired during free breathing for each participant in around 48 s.

### 2.3 Lung and Vessel Segmentation

**FIGURE 2:**
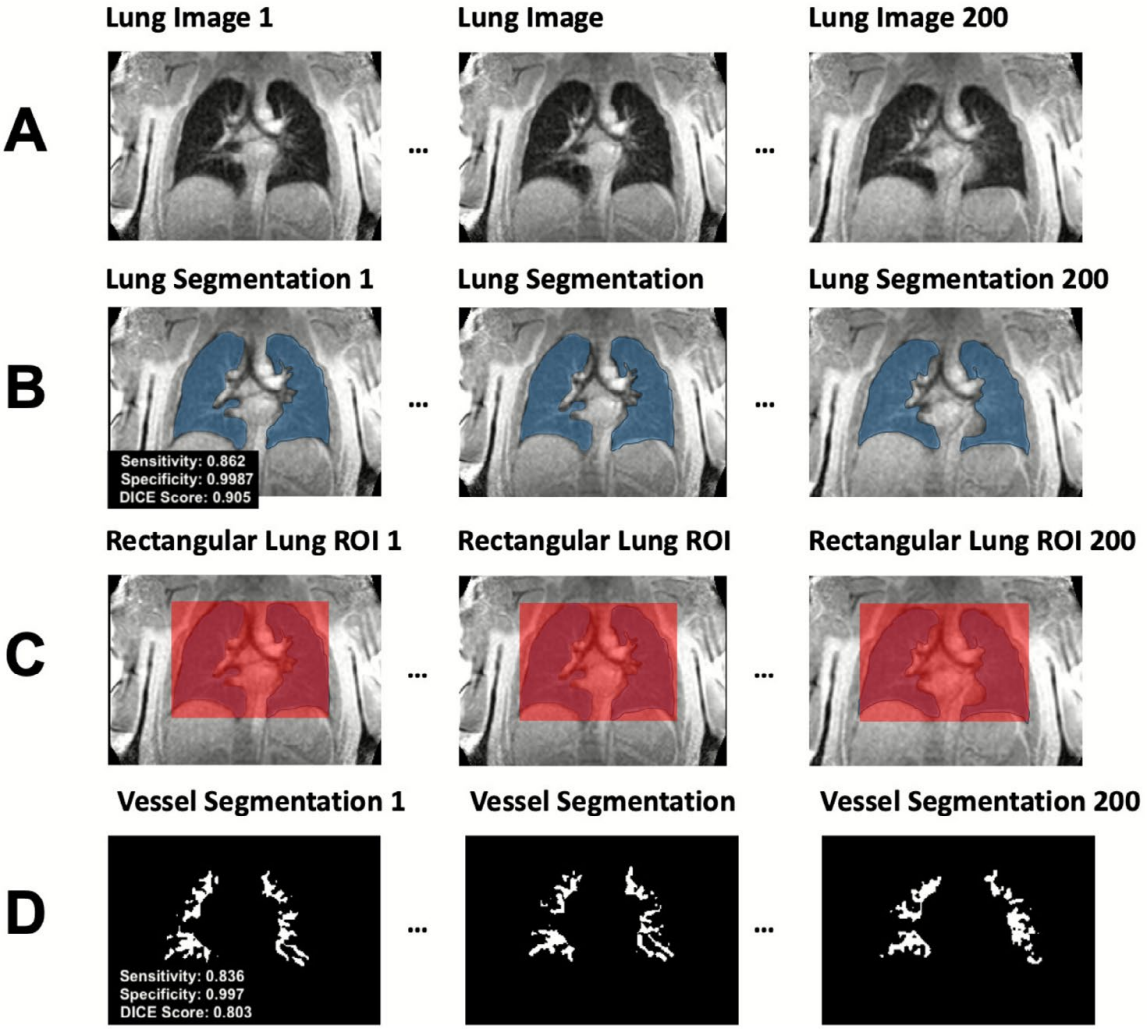
Illustration of the automated segmentation process. In (A), all lung images from each series were segmented by the U‐Nets. In the first step (B), lung segmentations excluding large central lung vessels were performed. Secondly, rectangular regions of interest (ROIs) within the outer boundaries of each lung segmentation were defined to avoid false vessel segmentations outside the thorax (C). Lastly (D), the vessel U‐Net segmentations were performed within the defined rectangular ROIs ranging from central to segmental arteries and veins.

In contrary to the signal‐oriented analysis method of phase‐resolved functional lung MRI (PREFUL) [1], no image registration was performed to maintain the spatial changes of the lung vessels throughout each series. For every frame, two 2D U‐Nets (Figure 2) were applied, which have been developed and validated previously, but have not been published explicitly [18, 22, 23, 24, 25].

The lung segmentation network was trained on a heterogeneous multi‐disease dataset. The distribution of patients across cohorts was as follows: CTEPH (24 training, 4 test), asthma (91 training, 20 test), CF (52 training, 9 test), COPD (262 training, 88 test), non‐CF lung disease (26 training, 6 test), and an additional cohort without specified diagnosis (100 training, 9 test). Overall, 3714 image slices were assigned to the training set and 1058 slices to the independent test set, corresponding to a test proportion of approximately 22%. Model optimization was performed exclusively on the training data using fivefold cross‐validation. The 3714 training slices were randomly divided into five equally sized folds. In each iteration, four‐folds were used for training and one‐fold for validation. The lung segmentation U‐Net achieved a sensitivity of 0.862, a specificity of 0.9987 and a Dice‐score of 0.905. To avoid false‐positive vessel segmentations outside the thorax, a rectangular region of interest (ROI) was automatically constructed within the outer boundaries of the lung segmentation. Within this ROI, the second 2D U‐Net segmented the pulmonary vessels (arteries and veins combined).

The vascular segmentation network was trained, validated, and tested on 272 manually segmented images from 57 different patients, that were previously examined at the institution. Diagnoses of these individuals included cystic fibrosis (seven patients), chronic thromboembolic pulmonary hypertension [6], and COPD [9], while most patients had no recorded diagnosis. Each patient contributed 1–12 images (mean 4.7). Data were split into training, validation, and test sets using a random assignment with a fixed random seed to ensure reproducibility. Images from the same patient were assigned exclusively to one subset to prevent data leakage, resulting in 217 training images (66 used for validation) and 55 test images. The U‐Net achieved a median sensitivity of 0.836, a median specificity of 0.9971 and a median DICE score of 0.803 against the expert ground truth. All segmentations were reviewed by a radiologist (JG) with 7 years of pulmonary MRI experience.

### 2.4 Statistical Analysis

The resulting averaged vessel signal per image and the vessel area of each image were plotted over time. A high‐pass filter with a cutoff frequency of 0.6 Hz was applied to remove effects associated with respiratory frequency. The coefficient of variation (CV; standard deviation divided by mean value) was reported as a percentage, indicating the relative variability of the measurements across the time series to ensure comparability across different cohorts.

**FIGURE 3:**
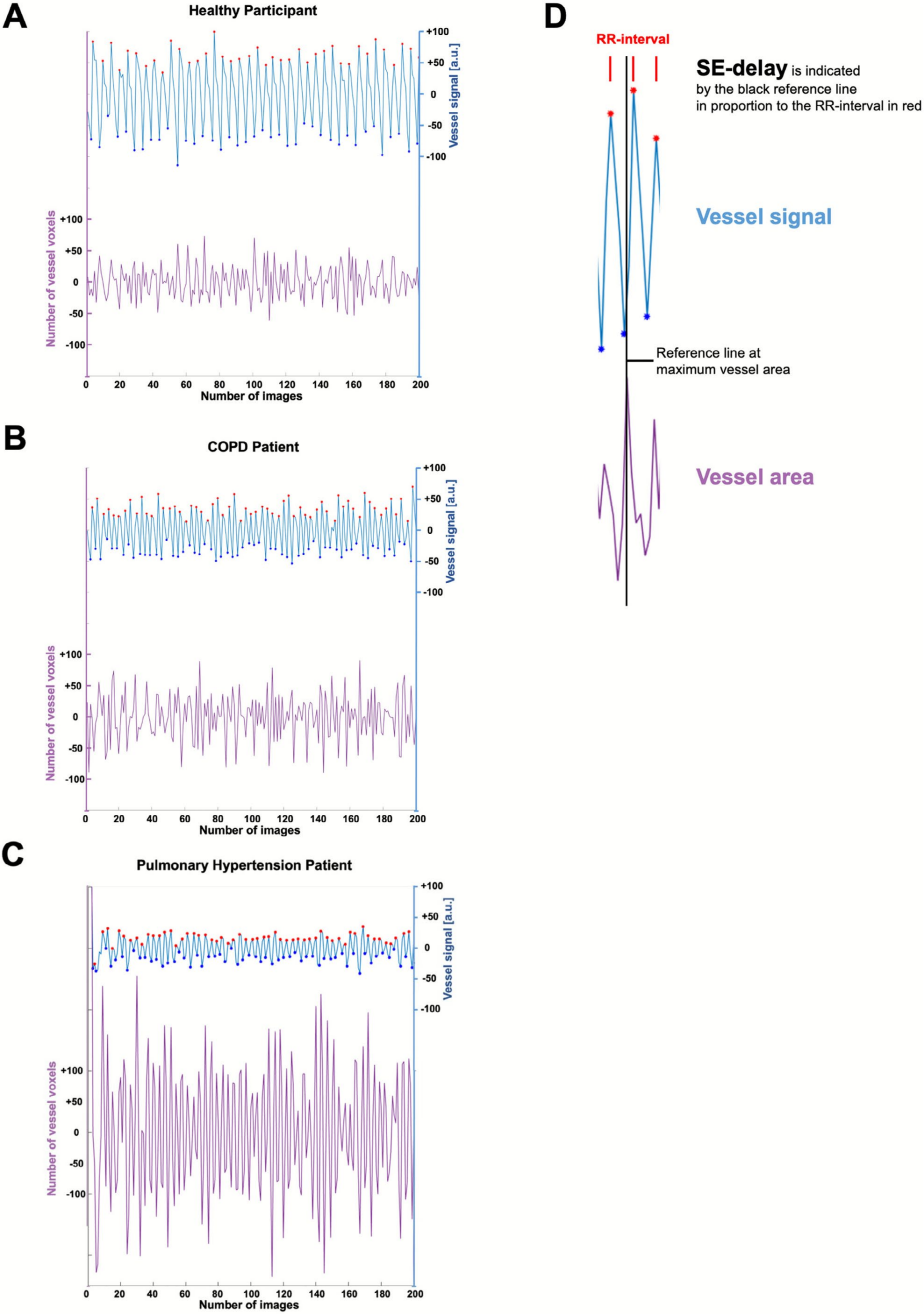
Graph showing the plotted vessel signal (blue) and the number of vessel voxels (violet) throughout the entire series of a healthy participant (19‐year‐old female), of a COPD patient (63‐year‐old female, GOLD = 2) and of a patient with postcapillary pulmonary hypertension (68‐year‐old male) in (A–C). In (C), the mean pulmonary artery pressure was 35 mmHg, pulmonary artery wedge pressure was 28 mmHg, cardiac output was 7.4 L/min, pulmonary vascular resistance was 76 dyn × sec × cm^−5^. Notice the decreasing amplitude of the vessel signal in both patients compared to a healthy participant and the large amplitude of the number of vessel voxels over time in the PH patient. Systolic (red) and diastolic (blue) timepoints were identified using a peak detection algorithm in the vessel signal. Values are centered around zero due to filtering. In (D), the calculation of the parameter SE‐delay is indicated by the black reference line in proportion to the RR‐interval. COPD, chronic obstructive pulmonary disease.

**FIGURE 4:**
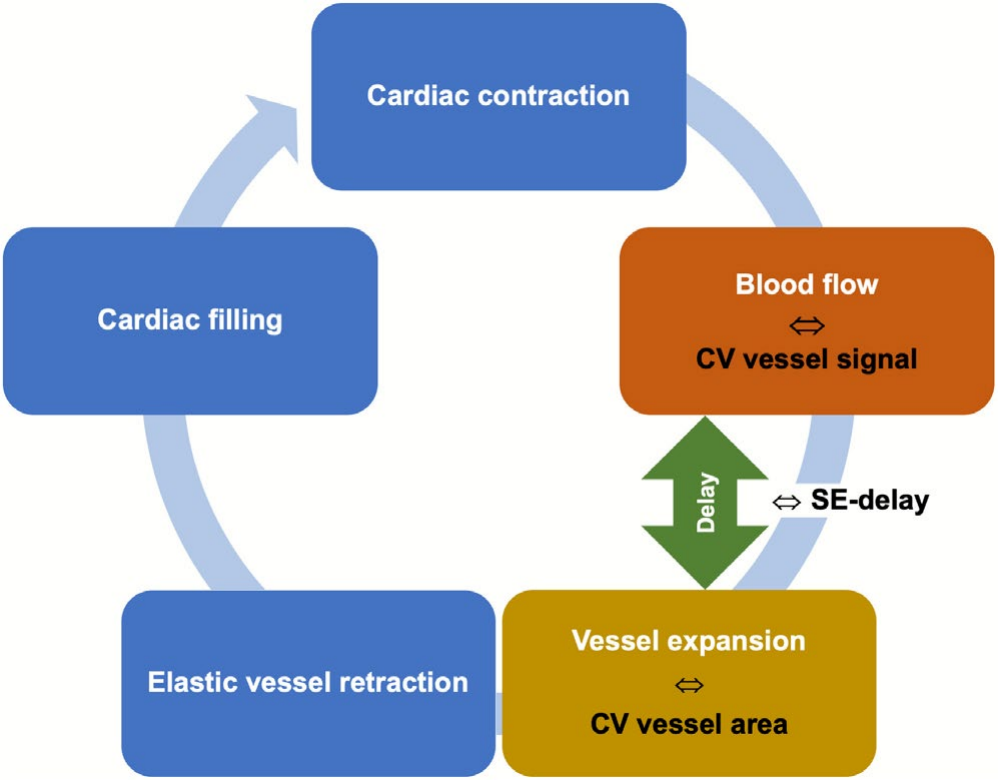
Schematic process of the cardiac cycle. The vessel flow is linked to CV vessel signal via flow‐related enhancement and the vessel expansion is represented by CV vessel area. Also, the delay between the maximum signal within the vessels (blood flow) and the maximum vessel area (expansion) was calculated, delivering SE‐delay in % of RR‐interval. CV, coefficient of variation.

Following parameters were considered:

- *CV vessel area*: Variability of the vessel voxel count as an indicator of the pulsatile vessel expansion. Calculated as SD of vessel voxel number divided by mean vessel voxel number.
- *CV lung area*: Variability of the lung voxel count as an indicator of the tidal breathing depth. Calculated as SD of lung voxel number divided by mean lung voxel number.
- *CV vessel signal*: Variability of the averaged vessel signal as an indicator of flow‐related signal enhancement. Calculated as SD of vessel voxel signal divided by mean vessel voxel signal.
- *Delay between signal and expansion in % of RR‐interval (SE‐delay)*: Average delay between the vessel signal and vessel area curves (Figures 3D and 4). A peak‐detection algorithm was used to identify the peaks in the vessel signal and vessel area plots. The delay from each signal peak to the next vessel area peak was calculated and then averaged across the series. The average delay was calculated in relation to the RR interval.
- *Heart rate*: In beats per minute based on the number of signal peaks.

Statistical analysis was performed using MATLAB R2024a (The MathWorks, Natick, MA, USA). Descriptive statistics were reported as median and interquartile range (IQR). The Shapiro–Wilk test was used to assess normality for all parameters. Because several parameters were not normally distributed, non‐parametric statistical tests were consistently applied.

Within the healthy cohort, intra‐scan and inter‐scan repeatability were evaluated using the Friedman test with Dunn‐Šidák post hoc comparisons. Repeatability metrics included two‐way random effects intraclass correlation coefficients (ICC (2,1)), which were interpreted as poor < 0.50, moderate 0.50–0.75, good 0.75–0.90 or excellent > 0.90 [26]. In addition, the standard error of measurement (SEM) and the minimal detectable change (MDC) for intra‐ and inter‐session comparisons were calculated. Bland–Altman plots of the parameters CV vessel area, CV vessel signal and SE‐delay were generated for intra‐ and inter‐scan comparisons.

To assess group differences between healthy participants, COPD patients, and PH patients, the Kruskal–Wallis test was used, followed by Dunn‐Šidák post hoc analysis for pairwise comparisons. Effect sizes were quantified using eta squared (*η* ^2^). The *η* ^2^ values were interpreted according to the classification of Cohen [27], where values between 0.01 and 0.06 were considered to indicate a small effect, values between 0.06 and 0.14 a moderate effect, and values greater than 0.14 a large effect.

To determine the independent contributions of demographic and anthropometric factors, multiple linear regression models were constructed for each imaging parameter. Cohort status (Healthy, COPD, PH), gender, age, and body surface area (BSA) were included as predictor variables. Body surface area formula of Dubois was applied.

*p*‐values were adjusted for multiple testing using the Bonferroni correction. Values ≤ 0.05 were considered statistically significant.

## 3 Results

### 3.1 Repeatability—Healthy Cohort

Across repeated acquisitions, CV vessel area, CV lung area, and CV vessel signal were overall stable (Friedman *p* = 0.950, *p* = 0.087, and *p* = 0.158, respectively). However, CV lung area decreased continuously across the 4 repetitive measurements without significance (*p* > 0.087). In contrast, SE‐delay differed significantly across acquisitions and was significantly higher in A1 than in subsequent scans with repositioning and new slice planning (A1 vs. B1 and A1 vs. B2), while intra‐session comparisons remained consistent. Similarly, the heart rate showed a small but significant reduction across sessions, with minimal intra‐session effects. Median (IQR) values for each metric are reported in Table 2. An example of the plotted signal height and voxel numbers for a healthy participant is provided in Figure 3A.

**TABLE 2:** Intra‐ and inter‐scan repeatability of vessel and lung parameters in the healthy cohort.

Parameter	Session A		Session B		Friedman *p*‐value	Dunn‐Šidák
	First scan (A1)	Second scan (A2)	First scan (B1)	Second scan (B2)		
CV vessel area (%)	2.97 [2.55–3.54]	3.04 [2.48–3.44]	2.98 [2.43–3.42]	3.06 [2.52–3.45]	0.950	n.a.
CV lung area (%)	8.41 [5.77–13.09]	8.15 [5.58–11.94]	7.65 [5.78–10.65]	6.85 [5.70–10.53]	0.087	n.a.
CV vessel signal (%)	4.02 [3.76–5.09]	4.08 [3.57–5.02]	4.28 [3.99–5.35]	4.46 [3.65–5.27]	0.158	n.a.
SE‐delay (% of RR‐interval)	62.97 [56.60–69.38]	58.00 [55.68–67.00]	58.26 [49.40–66.68]	53.69 [47.92–64.10]	**0.001**	A1 vs. B1: *p* = 0.013; A1 vs. B2: *p* = 0.001
Heart rate (min‐^1^)	78 [66–83]	71 [65–79]	70 [63–79]	71 [61–78]	**0.007**	A1 vs. B2: *p* = 0.011

*Note*: Values are median [IQR]. A1/A2 and B1/B2 denote the first and second repeated measurements within session A and session B, respectively. Significant *p*‐values are printed in bold.

Abbreviations: CV, coefficient of variation; SE‐delay, delay between signal and expansion.

Repeatability analysis confirmed moderate to good reliability for most dynamic parameters for intra‐session comparisons, with lower consistency for inter‐session comparisons. CV vessel area, CV lung area, CV vessel signal, and SE‐delay all showed higher ICC Intra values (0.71–0.83) compared with ICC Inter (0.26–0.86), indicating superior intra‐session stability. Correspondingly, SEM and MDC values were lower for intra‐scan than inter‐scan measurements, demonstrating higher precision when subjects remained in the scanner. The heart rate exhibited the highest reproducibility (ICC Intra = 0.89, ICC Inter = 0.61), confirming stable heart rate detection across acquisitions. Detailed SEM, MDC, and ICC values are summarized in Table 3.

**TABLE 3:** Intra‐ and inter‐scan repeatability parameters in the healthy cohort.

Parameter	SEM Intra	SEM Inter	MDC Intra	MDC Inter	ICC Intra	ICC Inter
CV vessel area (%)	0.74	1.32	2.05	3.66	0.83	0.26
CV lung area (%)	2.16	1.67	5.99	4.64	0.71	0.86
CV vessel signal (%)	0.54	0.56	1.5	1.55	0.75	0.64
SE‐delay (% of RR‐interval)	6.31	8.07	17.5	22.37	0.72	0.53
Heart rate (min‐^1^)	4	6	10	17	0.89	0.61

*Note*: Standard error of measurement (SEM), minimal detectable change (MDC), and two‐way random effects intraclass correlation coefficients (ICC (2,1)) are shown for intrascan (without leaving the scanner) and interscan (after repositioning) comparisons.

Abbreviations: CV, coefficient of variation; SE‐delay, delay between signal and expansion.

**FIGURE 5:**
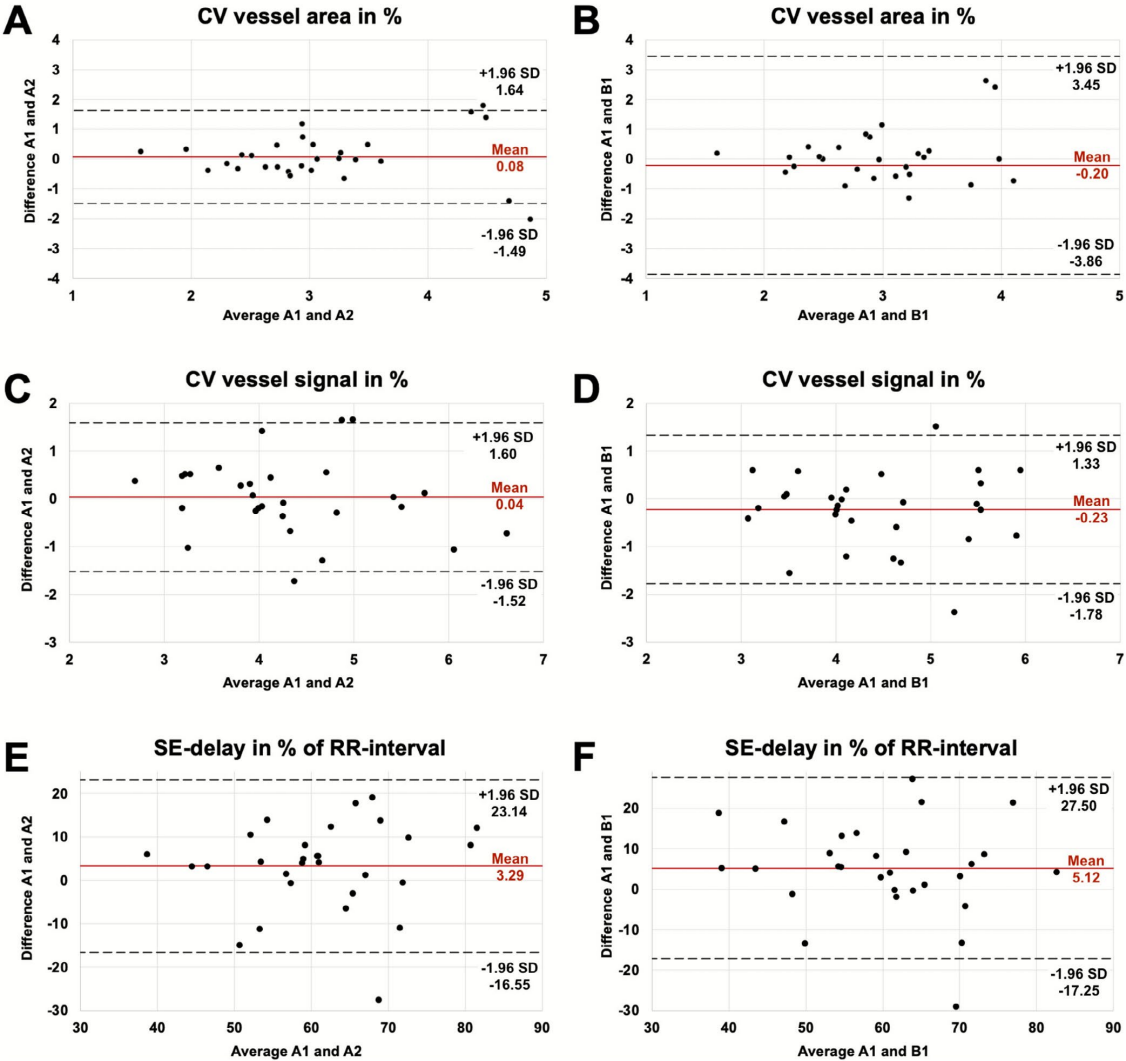
Bland–Altman plots illustrating intra‐session (A, C, E) and inter‐session (B, D, F) agreement for the functional parameters. Panels A and B show the agreement for CV vessel area, panels C and D for CV vessel signal, and panels E and F for SE‐delay. For each comparison, the mean difference (red line) and limits of agreement (dotted lines) are displayed. Notice the increasing limits of agreement in B. Nevertheless, one extreme outlier at −8 difference and 8 mean value is not depicted. CV, coefficient of variation; SE‐delay, delay between signal and expansion.

Both intra‐session and inter‐session Bland–Altman plots demonstrated no systematic bias, with differences centered around zero and no observable trend suggesting proportional or magnitude‐dependent errors (Figure 5).

### 3.2 Cohort Comparison

Significant age differences between healthy participants and COPD and PH patients. While the healthy and the COPD cohort comprise mainly men, a female dominance is present in the PH cohort.

Dynamic vascular and respiratory parameters showed distinct patterns among healthy participants, COPD patients, and PH patients. CV vessel area and CV lung area differed significantly between groups, with markedly increased variability in PH and reduced variability in COPD. Post hoc analysis confirmed significantly higher values in PH compared with both healthy participants and COPD patients, while healthy and COPD groups did not differ significantly. Effect sizes were large (*η* ^2^ = 0.35 and 0.37, respectively). CV vessel signal showed no significant group effect (*p* = 0.090). The temporal parameter SE‐delay was significantly prolonged in COPD (81.98%) compared to PH (69.87%) and to healthy subjects (62.97%), with all pairwise differences reaching significance except between healthy and PH (*p* = 0.223). The effect size was of comparable magnitude to that observed for CV vessel area and CV lung area (*η* ^2^ = 0.34). Heart rate also differed significantly between cohorts, showing the expected physiological trend of reduced values in COPD and elevated values in PH compared with healthy controls. The effect size was large (*η* ^2^ = 0.47). Median [IQR] values and statistical outcomes are summarized in Table 4 and Figure S1. Examples of the plotted signal height and voxel numbers for a patient with COPD and a patient with postcapillary PH are provided in Figure 3B,C.

**TABLE 4:** Group comparison of vessel and lung parameters between healthy participants and patients with COPD or pulmonary hypertension.

Parameter	Median healthy (A)	Median COPD (B)	Median PH (C)	Kruskal–Wallis *p*‐value	Dunn	*η* ^2^
CV vessel area (%)	2.97 [2.55–3.54]	2.67 [2.27–3.30]	4.58 [3.89–6.40]	**< 0.001**	A vs. B: *p* = 0.378; A vs. C: *p* < 0.001; B vs. C: *p* < 0.001	0.35
CV lung area (%)	8.41 [5.77–13.09]	7.03 [5.67–9.38]	16.74 [13.73–27.71]	**< 0.001**	A vs. B: *p* = 0.264; A vs. C: *p* < 0.001; B vs. C: *p* < 0.001	0.37
CV vessel signal (%)	4.02 [3.76–5.09]	3.64 [3.07–4.29]	3.68 [2.58–5.63]	0.090	n.a.	0.03
SE‐delay (% of RR‐interval)	62.97 [56.60–69.38]	81.98 [74.27–86.38]	67.20 [61.43–77.10]	**< 0.001**	A vs. B: *p* < 0.001; A vs. C: *p* = 0.223; B vs. C: *p* = 0.009	0.34
Heart rate (min^−1^)	78 [66–83]	65 [60–68]	85 [80–91]	**< 0.001**	A vs. B: *p* = 0.001; A vs. C: *p* = 0.025; B vs. C: *p* < 0.001	0.47

*Note*: A1 data from the repeatability test was used for the healthy cohort. Values are median [IQR]. Significant *p*‐values in bold.

Abbreviations: COPD, chronic obstructive pulmonary disease; CV, coefficient of variation; PH, pulmonary hypertension; SE‐delay, delay between signal and expansion.

### 3.3 Multivariable Regression

Multiple linear regression identified cohort status as the strongest independent predictor for most dynamic parameters after adjustment for gender, age and body surface area (BSA).

For CV vessel area, the opposite directional effects were confirmed (increased in PH, decreased in COPD; *F* = 9.98). No additional significant influence of gender, age, or BSA was observed (*p* ≥ 0.258). Also for CV lung area, cohort status again was the dominant effect (*F* = 30.16). BSA showed a modest but significant positive association, while gender and age were non‐significant (*p* ≥ 0.391). No variable significantly explained variance in CV vessel signal, confirming the absence of disease‐ or demographic dependence (*p* ≥ 0.069). For SE‐delay, COPD was the only disease category with a strong and independent prolongation (*F* = 4.62). Additionally, larger BSA was associated with shorter SE‐delays, whereas gender and age had no significant effects (*p* ≥ 0.079). For the heart rate, COPD was the only disease category with a strong and independent decrease and no demographic or anthropometric covariates have reached significance (*p* ≥ 0.139). Regression coefficients (Estimate ± SE), *t*‐ and *p*‐values, and overall predictor *F*‐values are provided in Table 5.

**TABLE 5:** Multiple linear regression analysis of independent effects of cohort status, gender, age and body surface area (BSA) on dynamic imaging parameters.

Outcome	Term	Estimate	Standard error	*t*	*p*	Term (effect)	*F*
CV vessel area (%)	Intercept	0.0105	0.0417	0.25	0.803		
	Status [PH]	−0.0128	0.0105	−1.22	0.227		
	**Status [COPD]**	−0.0116	0.0055	−2.1	**0.039**	Status	9.98
	Gender [F]	0.0042	0.0038	1.12	0.267	Gender	1.25
	Age	−0.0001	0.0004	−0.23	0.822	Age	0.05
	BSA	0.0187	0.0165	1.14	0.258	BSA	1.30
CV lung area (%)	Intercept	0.0185	0.0880	0.21	0.834		
	**Status [PH]**	−0.0507	0.0221	−2.29	**0.024**		
	**Status [COPD]**	−0.0406	0.0117	−3.48	**0.001**	Status	30.16
	Gender [F]	0.0057	0.0080	0.71	0.477	Gender	0.51
	Age	−0.0007	0.0008	−0.86	0.391	Age	0.74
	BSA	0.0740	0.0347	2.13	**0.035**	BSA	4.55
CV vessel signal (%)	Intercept	0.0397	0.0202	1.97	0.051		
	Status [PH]	0.0024	0.0051	0.47	0.638		
	Status [COPD]	−0.0035	0.0027	−1.29	0.201	Status	1.05
	Gender [F]	0.0034	0.0018	1.84	0.069	Gender	3.38
	Age	0.0000	0.0002	0.11	0.912	Age	0.01
	BSA	0.0007	0.0080	0.08	0.933	BSA	0.01
SE‐delay (%)	Intercept	1.0162	0.1901	5.35	< 0.001		
	Status [PH]	−0.0880	0.0478	−1.84	0.069		
	**Status [COPD]**	0.0757	0.0252	3	**0.003**	Status	4.62
	Gender [F]	−0.0307	0.0173	−1.78	0.079	Gender	3.16
	Age	0.0003	0.0016	0.17	0.864	Age	0.03
	**BSA**	−0.1596	0.0750	−2.13	**0.036**	BSA	4.53
Heart rate (min^−1^)	Intercept	88.2470	16.6450	5.3	< 0.001		
	Status [PH]	1.3170	4.1860	0.31	0.754		
	**Status [COPD]**	−13.1010	2.2080	−5.93	**< 0.001**	Status	34.04
	Gender [F]	−2.2600	1.5130	−1.49	0.139	Gender	2.23
	Age	0.0780	0.1429	0.55	0.586	Age	0.30
	BSA	−8.5380	6.5650	−1.3	0.196	BSA	1.69

*Note*: Regression coefficients (Estimate ± Standard Error), *t*‐ and *p*‐values, and overall predictor *F*‐values are provided. Significant *p*‐values and term in bold.

Abbreviations: COPD, chronic obstructive pulmonary disease; CV, coefficient of variation; PH, pulmonary hypertension; SE‐delay, delay between signal and expansion.

## 4 Discussion

In this feasibility study, an approach for quantitative dynamic vessel analysis using fully automatic 2D U‐Net segmentation in free‐breathing lung MRI was proposed. The study revealed significant differences in dynamic parameters such as the relative vessel area and signal variability and their respective phase delay.

Most parameters demonstrated lower interscan ICC values, which is likely the result of new slice planning after the break. While this predominantly affects the absolute level of the parameters, the relative ranking between individuals remained relatively good at demonstrating reliability for inter‐individual comparisons.

To ensure comparability between cohorts, functional parameters were compared based on their relative change by normalizing to the mean value. Especially for the signal, this was necessary due to the high dependence on factors such as patient positioning, coil sensitivity, or receiver gain. Although flow‐related enhancement is a widely documented source of signal variability and has been leveraged in many studies as an indirect marker of perfusion, this work did not focus on signal quantification [1, 28, 29, 30]. Rather, the characterization of vessel expansion and the examination how its timing relates to the corresponding flow‐driven signal fluctuations was pursued. Nevertheless, future studies could combine both features to achieve more comprehensive information. For instance, a correlation to ventilation parameters could identify affected lung areas and facilitate a (sub)segmental analysis and interpretation.

The large number of frames acquired at a relatively high temporal resolution (~300 ms) enabled detection of even minor differences among the cohorts although slightly differing imaging parameters could potentially have had an impact on the results. In general, the lung contains approximately 500 mL of circulating blood at any time, with cardiac output inducing a variation of ~50 mL per cardiac cycle (~10%) [31]. The measured pulsatile vessel area changes of 3%–5% fall within this physiologically plausible range.

Although physiological pulmonary vascular resistance is roughly one‐tenth of the systemic circulation, the pulmonary vasculature exhibits greater compliance, which decouples the right ventricle from the capillary bed. This buffering reduces pulse pressure, preserves capillary integrity, and enhances right heart efficiency—analogous to the “Windkessel” effect in the aorta. In this context, the SE‐delay may represent a surrogate marker for vascular elasticity or stiffness [32, 33, 34].

In the COPD cohort, a significantly longer phase SE‐delay may be influenced by the known vascular stiffening, elevated intrathoracic pressure from hyperinflation or structural degradation from emphysema [5]. The significant association between BSA and SE‐delay in the multiple linear regression may be caused by the larger vessel volume, which needs to be filled to reach maximum expansion. In the PH cohort, elevated mean pulmonary arterial pressure (mPAP), coupled with central vessel dilation and reduced compliance, may lead to faster vessel expansion with shorter SE‐delay [32]. However, due to the higher heart rate, this effect is canceled when considering the SE‐delay in relation to the RR. Both conditions could promote accelerated pulse wave reflections, increasing right ventricular afterload.

The reduced vessel expansion in COPD aligns with prior xenon‐MRI studies showing reduced capillary RBC oscillations, which are driven by stroke volume and the balance between pre‐ and postcapillary impedance. In postcapillary PH, increased cardiogenic RBC oscillations are consistent with diastolic reserve limitations during the cardiac cycle due to high postcapillary impedance. This results in a transient pulmonary capillary blood volume increase, as the right ventricular output temporarily exceeds that of the left ventricle [17, 35, 36]. Therefore, dynamic vessel analysis could be valuable in assessing treatment responses or in differentiating hemodynamic subtypes of PH—such as distinguishing precapillary PH with lower pulsatility.

Achieving similar insights as xenon MRI regarding capillary blood oscillations is promising. In contrast to xenon imaging, our approach requires no specialized equipment, gas, or other contrast agents, and is based on a standard, free‐breathing gradient echo sequence [37]. The resulting images can be used for further postprocessing, such as phase‐resolved functional lung MRI (PREFUL) or other proton‐based functional techniques [1]. The spatial dynamics extracted here provide complementary information to signal‐based perfusion measures, revealing different tendencies among disease cohorts.

A potential source of uncertainty in the interpretation of flow‐driven signal fluctuations is the reliance on vessel segmentation accuracy. Erroneous segmentations could, in principle, introduce artificial signal changes and vice versa. This could confound the assessment of vessel expansion–related effects. However, in the present study, a temporal delay between the signal fluctuations and the corresponding vessel expansion was consistently observed. This temporal offset argues against a direct influence of signal intensity changes on the segmentation outcome, as segmentation‐driven effects would be expected to manifest synchronously with signal variations.

Furthermore, minor differences in the imaging parameters between cohorts may influence some of the derived metrics. Differences in scanner hardware, particularly in gradient performance and available sequence implementations, necessitated the use of distinct acquisition parameters to achieve high temporal resolution and minimal TE while maintaining sufficient SNR. As the data were pooled retrospectively, it was not possible to harmonize the parameters prospectively across scanners. For example, through differences in temporal resolution, subtle contrast variations, or slight differences in total acquisition duration. The latter may interact with cohort‐specific differences in typical heart rates, as this can lead to systematically different numbers of cardiac cycles being sampled. Whether this introduces a systematic bias depends on the degree of parameter variability within the acquisition window (approximately 1 min) and remains to be evaluated. Nonetheless, part of the variability is likely mitigated by the applied amplitude normalization procedures and by the fact that the total number of sampled cardiac cycles was relatively high across all cohorts.

### 4.1 Limitations

This study is missing a reference standard for the calculated parameters. Future work should aim to reproduce results from hyperpolarized xenon MRI studies, which showed increased RBC oscillations in patients with pulmonary fibrosis [14]. Another option would be to compare the delay between maximum flow and maximum diameter of the pulmonary trunk in phase‐contrast MRI to the presented SE‐delay.

Another major limitation of this study is the age and gender imbalance across cohorts, which could influence functional and structural vascular characteristics. Vascular stiffening is a natural aging process, and an age‐matched healthy cohort might exhibit fewer differences compared to the PH cohort [38].

Another limitation is the indiscriminate measurement of arteries and veins together with the relatively low image resolution. This may lead to an overestimation of the vessel area due to inclusion of arteries, veins, and alveolar tissue and could further be influenced by the depth of breathing. Nevertheless, as the pulmonary arteries are exposed to most of the pressure changes, we expect that their expansion is predominantly depicted by our evaluation. The current spatial resolution limits direct detection of changes in microvessels affected by COPD and PH. Although vascular remodeling appears to propagate into measurable differences in larger vessels, higher‐resolution imaging could improve sensitivity and allow detection of smaller changes and a separate segmentation of arteries and veins.

While recent segmentation frameworks such as nnU‐Net have demonstrated strong performance across a wide range of medical imaging tasks, the present work employs a 2D U‐Net architecture as implemented in the established PREFUL processing pipeline. This choice was motivated by the extensive prior validation of the PREFUL framework and the need to maintain methodological consistency. Introducing a different segmentation architecture would have required a comprehensive revalidation of the entire pipeline, which was beyond the scope of this study. Nevertheless, the integration of more recent architectures, including nnU‐Net with both 2D and 3D configurations, represents a promising direction for future work and may further improve segmentation robustness and generalizability.

Furthermore, the current algorithm may still have inaccuracies in assigning each systolic peak in the signal and expansion curves. Although the simpler approach might be more robust, but a cross‐correlation method may reveal more subtle differences. A determination of the SE‐delay might be more precise using cross‐correlation, or by constructing a synthetic cardiac cycle like the approach used in PREFUL‐MRI. Future analyses should also evaluate, whether the SD‐delay occur systematically or, whether there is a sporadic component. Another issue could be inconsistent vessel detection by a differing degree of through plane motion by specific pulmonary diseases like COPD. Future studies should also assess reproducibility in diseased cohorts, as heterogeneity is a key characteristic of conditions such as COPD and cystic fibrosis.

## 5 Conclusion

Quantitative lung vessel analysis using free‐breathing MRI and U‐Net segmentation can differentiate specific hemodynamic characteristics in healthy individuals and patients with COPD and PH. This method shows potential as a sensitive and non‐invasive tool for disease detection, phenotyping, and treatment monitoring in pulmonary pathology.

## Funding

The authors have nothing to report.

## Conflicts of Interest

The authors declare no conflicts of interest.

## Supporting information


**Figure S1:** Box plots with group comparison of vessel and lung parameters between healthy participants and patients with COPD or pulmonary hypertension. Values are median [IQR]. COPD, chronic obstructive pulmonary disease; PH, pulmonary hypertension; SE‐delay, delay between signal and expansion.
