# Supplementary figures and images for: Analysis of Pulsatile Vessel Expansion in Healthy, COPD‐ and PH‐Patients Using Dynamic Vessel Segmentation in Free‐Breathing Lung MRI
Source: J Magn Reson Imaging. 2026 Jan 31;63(5):1420–32. doi: 10.1002/jmri.70249 (PMC13066536; doi:10.1002/jmri.70249)

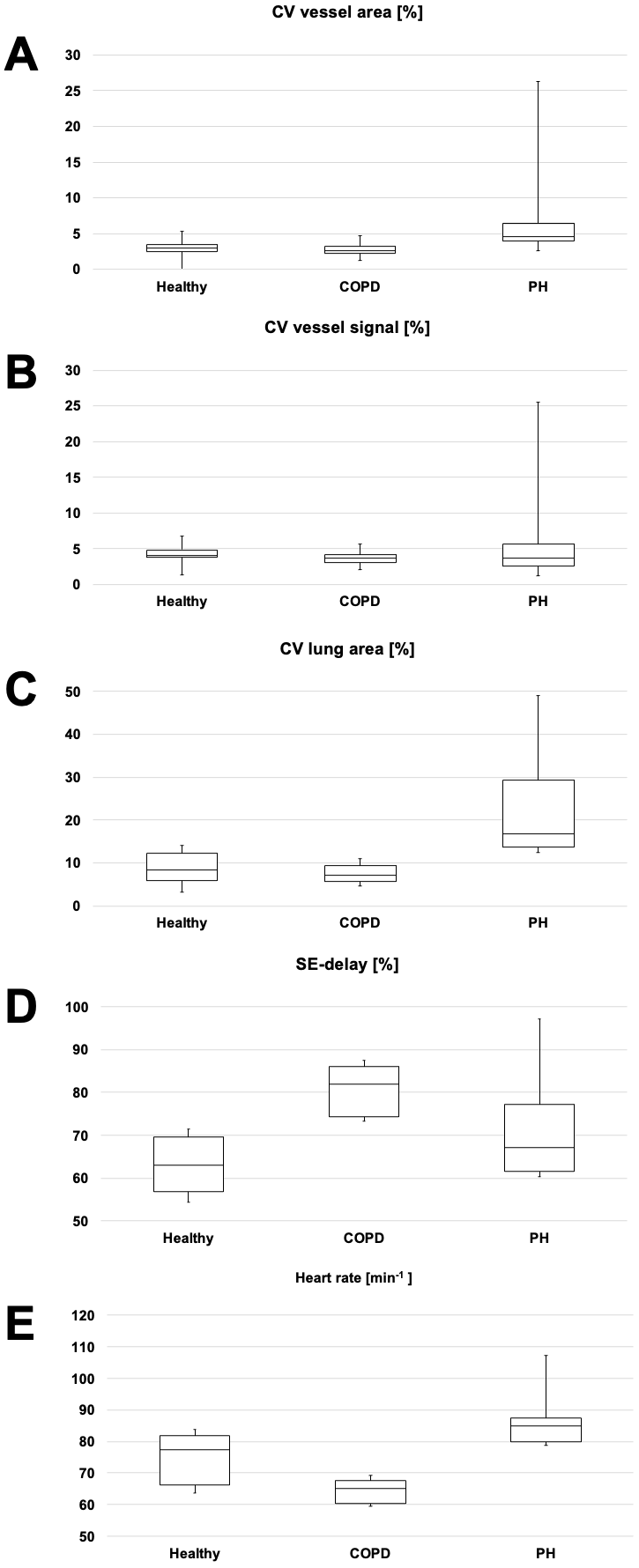

Supplement: Supplementary file 1 — Figure S1: Box plots with group comparison of vessel and lung parameters between healthy participants and patients with COPD or pulmonary hypertension. Values are median [IQR]. COPD, chronic obstructive pulmonary disease; PH, pulmonary hypertension; SE‐delay, delay between signal and expansion. [file JMRI-63-1420-s001.tiff]
